# Firmness at Harvest Impacts Postharvest Fruit Softening and Internal Browning Development in Mechanically Damaged and Non-damaged Highbush Blueberries (*Vaccinium corymbosum* L.)

**DOI:** 10.3389/fpls.2017.00535

**Published:** 2017-04-11

**Authors:** Claudia Moggia, Jordi Graell, Isabel Lara, Guillermina González, Gustavo A. Lobos

**Affiliations:** ^1^Plant Breeding and Phenomic Center, PIEI Adaptación de la Agricultura al Cambio Climático (A2C2), Universidad de TalcaTalca, Chile; ^2^Unitat de Postcollita-XaRTA, Centre AGROTÈCNIO, Universitat de LleidaLleida, Spain

**Keywords:** blueberry, bruising, soluble solids, acidity, maturity, firmness segregation, storage

## Abstract

Fresh blueberries are very susceptible to mechanical damage, which limits postharvest life and firmness. Softening and susceptibility of cultivars “Duke” and “Brigitta” to developing internal browning (IB) after mechanical impact and subsequent storage was evaluated during a 2-year study (2011/2012, 2012/2013). On each season fruit were carefully hand-picked, segregated into soft (<1.60 N), medium (1.61–1.80 N), and firm (1.81–2.00 N) categories, and then either were dropped (32 cm) onto a hard plastic surface or remained non-dropped. All fruit were kept under refrigerated storage (0°C and 85–88% relative humidity) to assess firmness loss and IB after 7, 14, 21, 28, and 35 days. In general, regardless of cultivar or season, high variability in fruit firmness was observed within each commercial harvest, and significant differences in IB and softening rates were found. “Duke” exhibited high softening rates, as well as high and significant *r*^2^ between firmness and IB, but little differences for dropped vs. non-dropped fruit. “Brigitta,” having lesser firmness rates, exhibited almost no relationships between firmness and IB (especially for non-dropped fruit), but marked differences between dropping treatments. Firmness loss and IB development were related to firmness at harvest, soft and firm fruit being the most and least damaged, respectively. Soft fruit were characterized by greater IB development during storage along with high soluble solids/acid ratio, which could be used together with firmness to estimate harvest date and storage potential of fruit. Results of this work suggest that the differences in fruit quality traits at harvest could be related to the time that fruit stay on the plant after turning blue, soft fruit being more advanced in maturity. Finally, the observed differences between segregated categories reinforce the importance of analyzing fruit condition for each sorted group separately.

## Introduction

Blueberry production has increased rapidly around the world over the last two decades (Lobos and Hancock, 2015). Chile is the second largest global producer, as well as the first exporter of fresh blueberries to the Northern Hemisphere (USA, Canada, Europe, and Asia). Most of the Chilean fruit is sent by boat, with transit periods of 20–50 days depending on destination. Blueberries are highly perishable, so fruit quality upon arrival to the final markets has major relevance to ensure economic returns (Beaudry et al., 1998; Retamales et al., 2014).

Several quality (dust, contaminants, size, bloom, russet/scars, attached stems, flower remains, and color) and condition (decay, mold, wounds, dehydration, firmness, and shriveling) traits are evaluated by inspection companies at destination markets. Among them, and regardless of season, dehydration and softening are the most common defects causing shipment rejections (Moggia et al., 2016b).

At present, due to low availability and high costs of labor for hand picking, farmers are being forced to invest in the mechanization of this critical production phase (Takeda et al., 2008; Xu et al., 2015). Mechanical harvesting of blueberries has the advantages of increasing capacity and efficiency as well as of reducing labor costs, but there are discrepancies as to their real contribution for the fresh fruit market. In general, machine harvest leads to the reduction of the acceptable amount of fruit that can be exported as a result of softening and excessive bruising; nevertheless, promising results have been reported on the use of a particular shaker, this being a viable alternative during critical periods (Lobos et al., 2014b). Fruit can also develop bruising during transport from the field to the packing-house, or when being processed on the packing-lines (Xu et al., 2015).

Blueberries are especially susceptible to mechanical damage, with injured berries resulting in loss of firmness that leads to reduced fruit quality and shelf-life (Xu et al., 2015). Bruises develop in the flesh of the damaged fruit as internal browning (IB) areas, resulting from tissue breakage and oxidation of phenolic compounds (Studman, 1997; Opara and Pathare, 2014). In order to relate the effect of mechanical damage with bruise damage, as done on large fruits and vegetables with instrumented spheres, a blueberry impact-recording device (BIRD) has been developed (Yu et al., 2011, 2014). Recently, Xu et al. (2015) measured the mechanical impacts on packing lines with the BIRD, showing that most of them occurred at the transfer points and that the highest impacts were recorded in one of the final handling steps, when the sensor dropped into the hopper above the clamshell filler.

Unfortunately, blueberry bruising can be expected to continue occurring, not only because of the use of mechanical/semi-mechanical harvest, or of differences between packing-line designs (e.g., number and height of transfer points, presence/absence of cushion materials), but also because of the lack of enough processing facilities during harvest peaks. Because of this, operators are forced to increase the speed at the sorting/packing lines, increasing the risk that fruit develop softening and IB during postharvest.

By simulating mechanical impact damage (as for other fruit species such as apples), the resistance of blueberries to IB has been evaluated by dropping fruit from different heights onto diverse surfaces; damage is rated on an internal bruise severity scale (affected area) after a period of cold storage (Brown et al., 1996; Yu et al., 2014). When berries were dropped from 15 to 30 cm onto hard surfaces, Brown et al. (1996) concluded that fruit developed IB on up to 50% of fruit area, and firmness declined significantly in samples having 25% or more damaged area. Yu et al. (2014) also reported a genotype effect, soft-textured cultivars being more susceptible than firm-textured ones when dropped on a hard plastic surface. However, all reported studies omit the high variability in firmness that occurs within a commercial clamshell, and hence the question arises whether results obtained for a given cultivar may be reproducible when variations in maturity stage, environmental conditions, and management procedures affect the proportions of soft, medium and firm fruit on a particular picking.

To the best of our knowledge, there are no previous reports on the implications of firmness segregation at harvest for the development of IB and softening of blueberries maintained under refrigerated conditions. Thus, the objective of this study was to understand how initial firmness and a single mechanical impact could affect the evolution of these traits during postharvest. For this, during two seasons, “Duke” and “Brigitta” fruit were segregated into soft, medium and firm categories at harvest, evaluating firmness loss and IB development of dropped (32 cm) and non-dropped fruit during 35 days under cold storage.

## Materials and Methods

### Plant Materials

During two consecutive seasons [2011/2012 (Y1) and 2012/2013 (Y2)], highbush blueberry (*Vaccinium corymbosum* L.) fruit of cultivars “Duke” and “Brigitta” (6- and 4-year-old, correspondingly) were collected at the peak of the commercial harvest from Chilean orchards located in Longaví (36°00′S; 71°35′W) and Santa Bárbara (37°29′S; 72°19′W), respectively. Both cultivars were planted on raised beds, at 3 m × 1 m in a loam soil. Each bed had two drip irrigation lines (2.4 L h^-1^ each 50 cm); irrigation frequency and timing were determined according to tensiometers established on each block at 30 and 50 cm depth. Pruning (May to July) was oriented to contribute for light entrance and air circulation, assuring a balance between canes of different ages and a stable production over time; pruning consisted in removing canes either unproductive or causing excessive shade on the plant. Fertigation was applied according to soil/foliar analysis and yield estimations; main nutrients were N (90–120 and 10–25 kg ha^-1^ for “Duke” and “Brigitta,” correspondingly), K_2_O (25–30 kg ha^-1^), and P_2_O_5_ (150–180 kg ha^-1^). Environmental conditions are summarized in Supplementary Table S1.

In order to mimic the marketable characteristics of exported fresh fruit, all fruit were harvested upon commercial criterion, which is based on 100% blue color (“Duke” December 5, 2011 and December 3, 2012; “Brigitta” December 29, 2011 and January 3, 2013). Berries were hand-picked by qualified workers belonging to each orchard. To avoid potential differences in sorting and packaging facilities, and to reduce IB damage, fruit were harvested directly into plastic clamshells (125 g). Fruit were immediately transported to the laboratory facilities at Universidad de Talca (35°24′S; 71°38′W), for further analysis and treatment establishment.

### Experimental Set-up and Measurements

Upon arrival to the research facilities, fruit were initially characterized in terms of firmness and IB, and then subjected to firmness segregation, impact damage simulation, and finally stored under refrigerated conditions as described below.

#### Firmness and IB at Harvest

**FIGURE 1:**
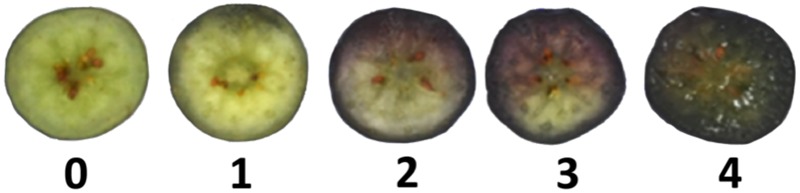
**Scale used for assessing internal browning (IB) severity in blueberry fruit.** Categories were assigned based on the extent of bruised equatorial area: 0 (0–5%), 1 (6–25%), 2 (26–50%), 3 (51–75%), and 4 (>75%).

In order to assess firmness and IB variability on commercial fruit coming from the field, a sample of 200 fruit were evaluated on each cultivar and season prior to firmness segregation. Firmness (N) was assessed using a compression device (FirmTech 2, BioWorks, KS, USA) with the force thresholds set between 200 g (maximum) and 15 g (minimum) (Ehlenfeldt and Martin, 2002; Saftner et al., 2008). IB was assessed by slicing fruit equatorially and then rating flesh browning on each individual fruit, according to the extent of the bruised area, as 0 (0–5%), 1 (6–25%), 2 (26–50%), 3 (51–75%), or 4 (>75%) (**Figure 1**).

#### Firmness Segregation and Initial Condition on Each Category

Using the same equipment as for firmness assessments, fruit were assigned to one of three firmness categories: soft (<1.60 N), medium (1.60–1.80 N), and firm (1.81–2.00 N). For each season, this segregation represented 50 clamshells (125 g) per cultivar and category, from which each replicate was withdrawn. Then, for each firmness group, the following traits were assessed as initial condition: (i) firmness on five replicates of 20 fruit each; (ii) total soluble solids (TSS, %) using a digital refractometer (Pocket PAL-1, Atago, Tokyo, Japan), from juice obtained from five replicates of five berries each; (iii) titratable acidity (TA, % citric acid equivalents) from five replicates; each one consisted of 10 mL of blueberry juice diluted to 100 mL with distilled water and titrated with 0.1 mol L^-1^ NaOH to an end-point pH of 8.2; (iv) TSS/TA ratio; and (v) IB on slices of five replicates of 20 fruit each.

#### Impact Damage Simulation

In order to study the evolution of IB and softening originated by impact damage, half of the fruit within each firmness category group were dropped from 32 cm onto a 30 cm × 30 cm of a hard plastic surface (6.4 mm-thick plexiglass), while the other half remained non-dropped. Dropping height was selected based on previous findings (data not published), as well as reports on extensive bruising resulting from 15–30 cm drop heights onto hard surfaces (Brown et al., 1996; Xu et al., 2015). For each cultivar, both dropped (32 cm) and non-dropped (0 cm) fruit were placed within clamshells into cardboard boxes, and then stored during 35 days at 0°C and 85–88% relative humidity (RH).

#### Firmness and IB Evolution during Postharvest

For each cultivar, firmness category group, and dropping treatment, firmness and IB evaluations were undertaken in samples (five replicates of 20 fruit each) from clamshells removed from cold storage after 7, 14, 21, 28, and 35 days. After each storage removal, fruit were acclimated to room temperature (18°C) for 3 h prior to perform measurements. Individual fruit were first assessed for firmness and then cut transversally for IB rating.

### Statistical Analysis

Firmness and IB condition of commercial fruit at harvest (before firmness segregation) was described for each cultivar and season, through box and whisker plots. Quality traits of fruit segregated at harvest were analyzed considering a completely randomized design with factorial arrangement, considering three firmness categories (soft, medium, and firm) × two seasons (Y1 and Y2). Data of parametric variables were subjected to analysis of variance (ANOVA), and significance of the differences was determined by Tukey’s test (*p* ≤ 0.05). IB data was subjected to non-parametric ANOVA with aligned rank for non-parametric analysis of multifactor designs (Oliver-Rodríguez and Wang, 2013) and mean separation by Tukey’s test (*p* ≤ 0.05) for ranked data.

For the postharvest study, in order to determine the relationships between firmness and IB during storage, data were subjected to regression analysis (*r*^2^) and models were fitted for each cultivar, season, firmness category, and drop heights. Additionally, statistical comparisons of slopes and intercepts between models for dropped vs. non-dropped fruit and, between firmness categories of each dropping treatment (soft vs. medium; medium vs. firm, and soft vs. firm) were performed. Data were transformed to obtain linearized models between firmness (x) and IB (y). The best-fitted model was 1/x for both cultivars. Analyses were executed using commercial statistical software Statgraphics Centurion XVI (v.16.0.09, Statpoint, VA, USA) and R 3.0.0 (R Development Core Team, 2008).

## Results

### Fruit Condition at Harvest

#### Firmness and IB before Fruit Segregation

**FIGURE 2:**
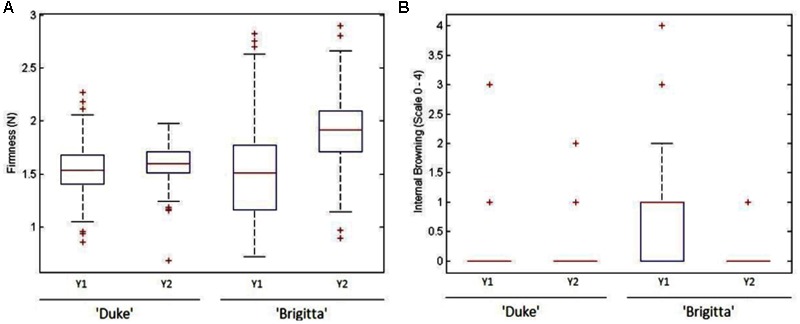
**Fruit firmness (A)** and internal browning **(B)** variability at commercial harvest of “Duke” and “Brigitta” blueberries, during seasons 2011/2012 (Y1) and 2012/2013 (Y2). IB categories: 0 (0–5%), 1 (6–25%), 2 (26–50%), 3 (51–75%), and 4 (>75%). *n* = 200 per year and cultivar. On each box and whisker plot, “+” represent outliers.

When commercial fruit sample was assessed for firmness at harvest, both cultivars displayed a wide range of values (**Figure 2A**). “Duke” firmness showed similar mean values during seasons 2011/2012 (Y1) and 2012/2013 (Y2) (1.55 and 1.60 N, respectively), whereas higher disparity was found on “Brigitta” (1.52 and 1.92 N, correspondingly). Yet, comparison by Kolmogorov–Smirnov test (*p* ≤ 0.05) evidenced significant differences in frequency distribution between years for both varieties (data not shown). Additionally, on both cultivars, fruit harvested on Y1 had greater variability (largest and smallest data values, wider quartile distributions, greater number of outliers) than berries picked on Y2. For “Duke,” 55 and 50% of fruit were below 1.6 N (upper threshold of the soft firmness category) for Y1 and Y2, respectively. For “Brigitta” these values reached 60 and 15% for Y1 and Y2, correspondingly. If a threshold of 1.4 N for very soft fruit is considered, 25% (Y1) and 10% (Y2) of “Duke” fruit were below that level, whereas values for “Brigitta” were 42 and 5% for Y1 and Y2, in that order (**Figure 2**).

Although not subjected to the dropping procedure, fruit displayed some IB at harvest (**Figure 2B**), with mean IB scores of 0.15–0.19 for “Duke,” and 0.10–1.01 for “Brigitta,” on Y1 and Y2, correspondingly. The most heterogeneous IB values were found for “Brigitta” fruit harvested on Y1. Yet, overall percentages of non-bruised fruit (category 0) at harvest were higher for “Brigitta” (90.0–92.5%) than for “Duke” (83.1–89.8%) (data not shown).

#### Fruit Quality after Firmness Segregation

Once samples were segregated by firmness, the ANOVA proved that fruit quality at harvest was influenced by initial firmness (**Table 1**). On both cultivars, firmer fruit was related to higher TA but lower TSS/TA and IB; TSS were significant only on “Brigitta,” and higher on the softer group (<1.60 N). Differences between years occurred for TSS, TA, and TTS/TA for “Duke” and for TSS, TA, and IB on “Brigitta,” reinforcing the higher variability found on this last trait during Y1. Significant interactions occurred for TA on “Duke” (with differences between categories on Y1, but no differences on Y2) and for IB on “Brigitta” (with differences only on soft fruit between years, having Y1 higher IB than Y2) (Supplementary Figure S1).

**Table 1:** Analysis of variance for fruit quality traits^a^ at harvest of “Duke” and “Brigitta” blueberries according to three-firmness category groups, during seasons 2011/2012 (Y1) and 2012/2013 (Y2).

Cultivar	Factor	TSS (%)	TA (% citric acid)	TSS/TA	IB (scale 0–4)
“Duke”	Firmness category (F)				
	Soft (<1.60 N)	11.2	0.59c	19.4a	0.38a
	Medium (1.60–1.80 N)	11.1	0.71b	17.5ab	0.44a
	Firm (1.81–2.00 N)	11.1	0.83a	15.1b	0.19b
	Year (Y)				
	Y1	12.4a	0.93a	13.7b	0.34
	Y2	9.9b	0.49b	20.9a	0.31
	Significance (*p*-value)				
	F	0.974^b^	0.000	0.049	0.000
	Y	0.000	0.000	0.000	0.069
	F × Y	0.527	0.002	0.118	0.352
“Brigitta”	Firmness category (F)				
	Soft (<1.60 N)	15.2a	0.57b	28.4a	1.28a
	Medium (1.60–1.80 N)	13.5b	0.64ab	21.4b	0.26b
	Firm (1.81–2.00 N)	13.2b	0.75a	18.0b	0.19c
	Year (Y)				
	Y1	15.1a	0.74a	22.3	0.69a
	Y2	12.8b	0.57b	22.9	0.45b
	Significance (*p*-value)				
	F	0.008	0.030	0.002	0.000
	Y	0.000	0.007	0.692	0.000
	F × Y	0.243	0.329	0.844	0.000

*For a given cultivar, or factor, and significance *p* ≤ 0.05, different letters within a column represent significant differences (Tukey’s test, *p* ≤ 0.05).*

*^a^Traits: total soluble solids (TSS), titratable acidity (TA), and internal browning (IB) damage categories: 0 (0–5%), 1 (6–25%), 2 (26–50%), 3 (51–75%), or 4 (>75%).*

*^b^In red, p-values lower than 0.05.*

### Firmness and IB Evolution of Dropped and Non-dropped Fruit during Postharvest

**FIGURE 3:**
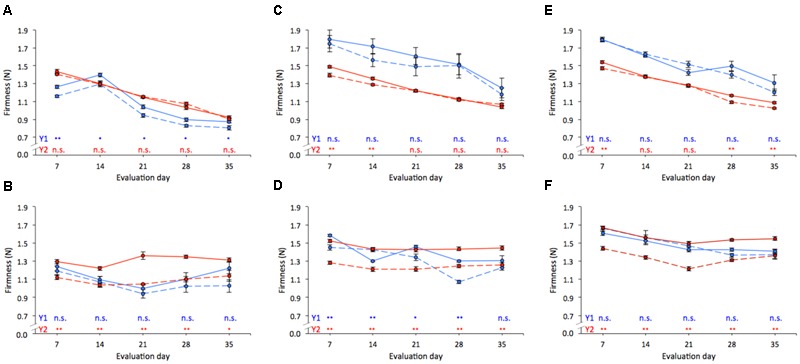
**Firmness (N) changes during refrigerated storage [0°C and 85–88% relative humidity (RH)] of “Duke” (A,C,E)** and “Brigitta” **(B,D,F)** blueberries, according to firmness segregation at harvest: soft (<1.60 N; **A,B**), medium (1.61–1.80 N; **C,D**), and firm (1.81–2.00 N; **E,F**). Assessments were taken during 2011/2012 and 2012/2013 (Y1, Y2; blue and red lines, respectively) on dropped (32 cm, dashed lines) and non-dropped (0 cm, solid lines) fruit. Each value represents the mean of five replicates of 20 fruit. Significance: n.s. (non-significant), ^∗^*p* < 0.05, ^∗∗^*p* < 0.01.

In comparison to “Brigitta,” “Duke” berries showed lower firmness retention along time, irrespective of firmness category, dropping treatment or season (**Figure 3**). Between harvest and the end of storage, and for both seasons, firmness of “Duke” blueberries was reduced on average by 39.8, 33.6, and 38.6% (**Figures 3A,C,E**) for soft, medium, and firm fruit, respectively (data not shown), whereas firmness loss in “Brigitta” averaged 17.3, 24.4, and 23.8%, correspondingly (**Figures 3B,D,F**). When dropped and non-dropped fruit were compared, “Brigitta” fruit appeared to be more sensitive to initial firmness, since significant differences between damaged and non-damaged fruit were found for most of storage evaluations (medium on Y1; soft, medium, and firm on Y2). In contrast, for “Duke” samples consistent differences between dropped and non-dropped fruit along the whole storage period were observed on soft fruit harvested on Y1 uniquely. Additionally, the magnitude of the differences between dropped and non-dropped fruit, as well as between seasons, were higher for “Brigitta.”

**FIGURE 4:**
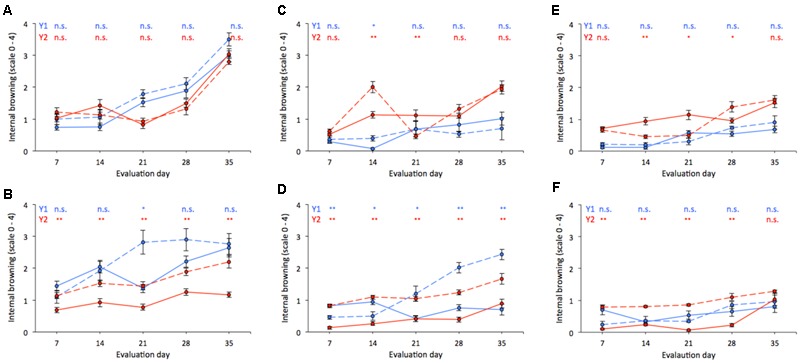
**Internal browning changes during refrigerated storage (0°C and 85–88% RH) of “Duke” (A,C,E)** and “Brigitta” **(B,D,F)** blueberries, according to firmness segregation at harvest: soft (<1.60 N; **A,B**), medium (1.61–1.80 N; **C,D**), and firm (1.81–2.00 N; **E,F**). Assessments were taken during 2011/2012 and 2012/2013 (Y1, Y2; blue and red lines, respectively) on dropped (32 cm, dashed lines) and non-dropped (0 cm, solid lines) fruit. Each value represents the mean of five replicates of 20 fruit. IB scale: 0 (0–5%), 1 (6–25%), 2 (26–50%), 3 (51–75%), and 4 (>75%). Significance: n.s. (non-significant), ^∗^*p* < 0.05, ^∗∗^*p* < 0.01.

In general, IB was higher after storage than at harvest, particularly for soft fruit (**Figure 4**), regardless of cultivar, year, or dropping treatment. “Duke” fruit exhibited relatively low IB values up to 21 days of storage, with the highest IB at 35 days for soft (Y1 and Y2) and medium firmness fruit (Y2) (**Figures 4A,C**). Similarly to the evolution of firmness in postharvest (**Figure 3**), “Duke” fruit also developed less IB in response to dropping, given that no significant differences between treatments were found at most of the evaluation dates. “Brigitta,” on the other hand, showed marked differences in IB development between dropped and non-dropped fruit for all firmness categories (**Figures 4B,D,F**). Compared to “Duke” and regardless of dropping treatment, “Brigitta” fruit developed lower IB within medium and firm categories (**Figures 4B,D**).

### Relationship between IB and Firmness

**FIGURE 5:**
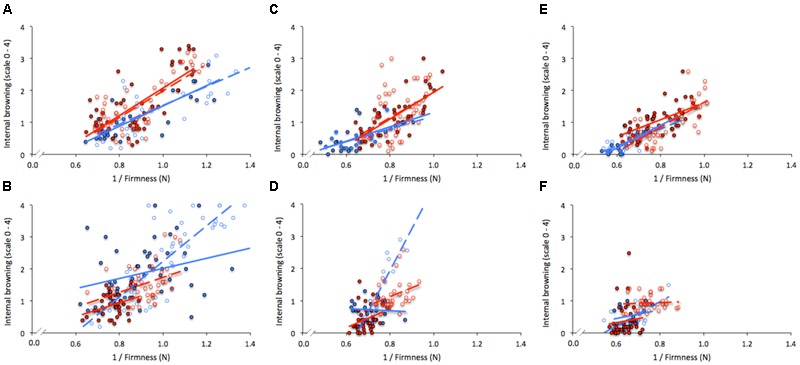
**Regression analysis between firmness (N) and internal browning for “Duke” (A,C,E)** and “Brigitta” **(B,D,F)** blueberries, according to firmness segregation at harvest: soft (<1.60 N; **A,B**), medium (1.61–1.80 N; **C,D**), and firm (1.81–2.00 N; **E,F**). Assessments were taken during 2011/2012 and 2012/2013 (Y1, Y2; blue and red lines, respectively) on dropped (32 cm, dashed lines) and non-dropped (0 cm solid lines) fruit. Each value represents individual fruit. IB scale: 0 (0–5%), 1 (6–25%), 2 (26–50%), 3 (51–75%), and 4 (>75%).

For “Duke” samples, the regression analyses (*r*^2^) between IB and firmness (**Table 2** and **Figure 5**) revealed significant effects on dropped and non-dropped fruit for all three firmness categories and for both seasons. Although *r*^2^ varied among comparisons, soft and firm fruit showed in general the highest values. In contrast, 9 out of the 12 models fitted for “Brigitta,” which included all non-dropped fruit of both years and dropped fruit of Y2, showed no significant associations. During Y1, the highest *r*^2^ values for dropped fruit were found on soft and medium fruit of this cultivar (72.7 and 80.6, respectively). The comparisons of slopes and intercepts between dropping treatments (**Table 2**) showed that significant differences for “Duke” were found only between intercepts of firm fruit harvested in Y2. In contrast, equations developed for “Brigitta” differed in slopes (soft and medium fruit of Y1) and intercepts (medium fruit of Y1, all three categories on Y2) on five out of the six instances.

**Table 2:** Internal browning (IB) vs. firmness (F) regression analysis for non-dropped (0 cm) and dropped (32 cm) fruit.

Cultivar	Year	Firmness category	Model			Model comparisons (*p*-values)	
			Equation	*n*^a^	*r*^2b^	Intercept	Slope
“Duke”	Y1	Soft (<1.60 N)	IB_0_ = -1.669 + 3.188 × (1/F)	30	69.2^∗∗∗^	0.772^c^	0.740
			IB_32_ = -1.429 + 2.966 × (1/F)	30	53.7^∗∗∗^		
		Medium (1.60–1.80 N)	IB_0_ = -1.213 + 2.745 × (1/F)	16	25.7^∗^	0.643	0.600
			IB_32_ = -0.796 + 1.994 × (1/F)	16	34.3^∗^		
		Firm (1.81–2.00 N)	IB_0_ = -1.936 + 0.825 × (1/F)	18	72.1^∗∗∗^	0.585	0.369
			IB_32_ = -1.365 + 2.711 × (1/F)	20	44.5^∗^		
	Y2	Soft (<1.60 N)	IB_0_ = -2.233 + 4.311 × (1/F)	50	50.3^∗∗∗^	0.528	0.864
			IB_32_ = -2.185 + 4.173 × (1/F)	50	58.0^∗∗∗^		
		Medium (1.60–1.80 N)	IB_0_ = -2.256 + 4.203 × (1/F)	50	55.0^∗∗∗^	0.747	0.834
			IB_32_ = - 2.013 + 3.953 × (1/F)	50	20.3^∗^		
		Firm (1.81–2.00 N)	IB_0_ = -1.039 + 2.656 × (1/F)	50	35.3^∗∗∗^	0.002	0.118
			IB_32_ = -2.265 + 3.899 × (1/F)	50	59.7^∗∗∗^		
“Brigitta”	Y1	Soft (<1.60 N)	IB_0_ = 0.439 + 1.584 × (1/F)	48	8.57^n.s.^	0.814	0.000
			IB_32_ = -3.333 + 5.576 × (1/F)	48	72.7^∗∗∗^		
		Medium (1.60–1.80 N)	IB_0_ = 0.963 – 0.340 × (1/F)	20	0.57^n.s.^	0.016	0.000
			IB_32_ = -8.393 + 12.921 × (1/F)	20	80.6^∗∗∗^		
		Firm (1.81–2.00 N)	IB_0_ = -0.359 + 1.354 × (1/F)	26	4.48^n.s.^	0.507	0.219
			IB_32_ = -2.060 + 3.798 × (1/F)	26	43.9^∗∗^		
	Y2	Soft (<1.60 N)	IB_0_ = -0.780 + 2.153 × (1/F)	40	12.4^n.s.^	0.028	0.904
			IB_32_ = -0.597 + 2.338 × (1/F)	40	11.7^n.s.^		
		Medium (1.60–1.80 N)	IB_0_ = -1.580 + 2.910 × (1/F)	40	7.06^n.s.^	0.012	0.947
			IB_32_ = -1.081 + 2.763 × (1/F)	40	10.6^n.s.^		
		Firm (1.81–2.00 N)	IB_0_ = -0.444 + 1.280 × (1/F)	28	0.97^n.s.^	0.000	0.609
			IB_32_ = 0.757 + 0.244 × (1/F)	28	0.36^n.s.^		

*^a^Sample size.*

*^b^Significance: n.s. (non-significant), ^∗^*p* < 0.05, ^∗∗^*p* < 0.01, ^∗∗∗^*p* < 0.001.*

*^c^In red, p-values lower than 0.05.*

*Intercept and slope comparison between IB_0_ and IB_32_, of “Duke” and “Brigitta” blueberries according to three firmness category groups, during seasons 2011/2012 (Y1) and 2012/2013 (Y2).*

When firmness categories were contrasted within the same dropping treatment (**Table 3**), outcomes varied among seasons. On non-dropped fruit of Y1, three comparisons resulted on different intercepts (medium vs. firm on “Duke”; soft vs. medium, and soft vs. firm on “Brigitta”), but no differences were found between slopes. For the same treatment, differences on Y2 occurred amid slopes of “Duke” (medium vs. firm, and soft vs. firm) and intercepts of “Brigitta” (soft vs. medium). Within dropped fruit of Y1 no significant differences were found for any comparison on “Duke,” whereas two cases were statistically significant for “Brigitta” (soft vs. medium differed on intercept and slope; medium vs. firm differed on slopes). On Y2, differences between intercepts of medium vs. firm, and soft vs. firm occurred for “Duke,” meanwhile for “Brigitta” the only significant difference happened between slopes of soft vs. firm fruit.

**Table 3:** Intercept and slope comparisons of internal browning vs. firmness regression analysis, between firmness category groups for non-dropped (0 cm) and dropped (32 cm) “Duke” and “Brigitta” blueberries, during seasons 2011/2012 (Y1) and 2012/2013 (Y2).

Cultivar	Drop height (cm)	Firmness category	Model comparisons (*p*-values)			
			Y1		Y2	
			Intercept	Slope	Intercept	Slope
“Duke”	0	Soft vs. medium	0.273^a^	0.746	0.304	0.902
		Medium vs. firm	0.035	0.572	0.779	0.045
		Soft vs. firm	0.840	0.783	0.152	0.066
	32	Soft vs. medium	0.911	0.517	0.908	0.851
		Medium vs. firm	0.164	0.492	0.007	0.702
		Soft vs. firm	0.576	0.877	0.001	0.870
“Brigitta”	0	Soft vs. medium	0.000	0.443	0.019	0.691
		Medium vs. firm	0.148	0.399	0.678	0.558
		Soft vs. firm	0.007	0.954	0.126	0.689
	32	Soft vs. medium	0.029	0.002	0.397	0.810
		Medium vs. firm	0.346	0.000	0.148	0.081
		Soft vs. firm	0.783	0.370	0.146	0.034

*^a^In red, p-values lower than 0.05.*

## Discussion

The analysis of fruit characteristics at harvest revealed two important aspects that have not been reported previously. The first one is that, regardless of cultivar or season, high variability in fruit firmness occurred within each commercial harvest. In comparison with other fruit species such as apple, for which very soft fruit (58–62 N) represent less than 0.5–0.8% (Herregods and Goffings, 1993; De Silva et al., 2000), a high percentage of “Duke” and “Brigitta” blueberries showed this characteristic (<1.4 N) in Y1 (25 and 42%, respectively) and Y2 (10 and 5%, respectively). The second one refers to the noticeable differences in quality traits found between firmness categories, which highlights the relevance of analyzing the development of softening and IB for each sorted group separately. These two aspects will be covered during the discussion.

### Susceptibility of Blueberries to Develop IB

IB was detected at harvest in this study, even though fruit were carefully hand-picked and not subjected to sorting or packing. Gołacki et al. (2009) indicated that vibration forces, usually occurring during transportation from the field, are difficult to avoid and may also cause damage. In addition to possible damage sources before harvest (e.g., due to wind or machinery), fruit samples used herein underwent a ∼3-h trip from the field to the laboratory, and hence transportation may have impacted the basal IB found. Indeed, unless a packinghouse facility is available at the producing orchard, it is common that fruit travel 2–3 h until being processed. This observation highlights the importance of careful handling of the fruit throughout the whole production and distribution chain, and evidences high differences within a particular cultivar among seasons. In fact, variability in firmness and IB at harvest showed dissimilarities between cultivars, with “Duke” fruit being more homogeneous for both seasons, whereas “Brigitta” berries showed higher differences within and between years. The high IB values in Y1 at harvest for “Brigitta” were associated to softer fruit (**Figure 2**). Variations in ambient temperature between both seasons (Supplementary Table S1) may partially account for the differences in fruit condition between seasons and cultivars, especially for higher heterogeneity of “Brigitta” samples on Y1. Although there is not much information, it has been suggested that an ideal range of temperatures for northern highbush blueberries might range 20–25°C (Davies and Flore, 1986); values above 30°C (also associated with high light intensity as in Chile) cause plant damage (Trehane, 2004; Lobos and Hancock, 2015), as well as lowered wax coverage of fruit, which tend to be smaller and softer (Mainland, 1989). With the exception of precipitation (Y1: 32.9 mm and Y2: 102 mm), Longaví does not usually register substantial differences in environmental conditions from early October (full bloom) to early December (harvest) (Supplementary Table S1). This might in part explain the lower variability between seasons observed for “Duke.” On the other hand, different temperature patterns for each season were registered in Santa Bárbara in December. Even though more favorable temperatures occurred in Y1 (20–25°C), more temperature extremes took place (greater number of hours or days hotter than 27, 29, and 32°C), probably leading to early softening of fruit.

It is also highly likely that blueberries can be damaged on packing-lines. Xu et al. (2015) studied 11 commercial packing lines using the BIRD and found that the tested lines differed in their combinations and alignments, thus creating different points for potential impact damage. Yet, all the impacts occurred at transfer points, the highest drop heights being 35–36 cm. Additionally, the latter part of the packing line, where fruit drop into the hopper for loading clamshells, is another point for potential damage due to the combination of hard contact surface (usually stainless steel) and high drop height (Xu et al., 2015), and especially when the first berries drop into the hopper, since they will impact directly onto the hard surface. As more fruit get into the line, ever more fruit-to-fruit impacts will take place, this being a source of impact that has not been fully incorporated in studies dealing with mechanical damage. Results obtained in the present study show that significant differences in IB development between “Duke” and “Brigitta” occurred with drop heights of 32 cm, evidencing a differential effect of season, cultivar, and firmness category.

In order to standardize sorting/packing-lines and to establish some basic recommendations to improve condition, it is critical to identify which fruit would be more prone to softening and IB during postharvest. Unfortunately, given that the main criterion for establishing harvest date of blueberries is skin color, and that high labor costs are associated to this operation (Brown et al., 1996; Takeda et al., 2008; Lobos et al., 2014b), growers wait for blue fruit to accumulate in the bush before starting commercial pickings. This practice results in fruit with similar external appearance but, as found in the present study, with important heterogeneity in maturity status, that will lead to a wide range of firmness levels at harvest, as well as in softening rates during postharvest. Previous works have proved that delaying harvest increases TSS and TSS/TA but reduces TA and firmness (Woodruff et al., 1960; Ballinger et al., 1963; Kushman and Ballinger, 1963; Lobos et al., 2014a), since TSS increase and acids decrease due to fruit respiration in the course of maturation (Famiani et al., 2005; Dai et al., 2009). In fact, when fruit showing no differences in skin color at harvest (determined either visually or instrumentally) were picked 2 or 6 days after turning 100% blue on the bush, important differences in fruit condition were demonstrated associated to these two maturity stages (Moggia et al., 2016a,b). In those previous studies, when similar percentages of green and pink fruit were reached early in the season, clusters with similar characteristics and canopy position were selected and labeled. Fruit development was followed until both maturity stages were reached: 100% blue and residing on the plant for a maximum of 2 days (ripe), and 100% blue and residing on the plant for 6 days (overripe). That methodology allowed the authors to conclude that, when these two maturity stages were selectively picked, important differences were found, “Duke” being more sensitive than “Brigitta” to this factor. The elapsed time between harvests was enough to increase TSS and TSS/TA of “Duke” samples, and to reduce fruit firmness in both cultivars. These findings reinforce the importance of the time that fruit stay on the plant after turning 100% blue for fruit heterogeneity. In the present study, segregation by firmness at harvest revealed similar trends for these traits, suggesting that fruit within the soft category had actually stayed longer in the plant after turning completely blue. Accordingly, when fruit were segregated based on firmness, berries assigned to the soft category displayed the highest IB, TSS, and TSS/TA values (**Table 1**). Given the variability found at harvest (box and whisker plots), these dissimilarities would be higher for Y1 “Brigitta” fruit, thus accounting for the greater differences found according to the dropping treatment between fruit within the soft and the medium categories. In fact, according to the Chilean blueberry industry, overall commercial defects (including softening, dehydration, and mechanical damage) differ between seasons, and the affected produce may account for 10–45% of the fresh fruit reaching final markets (Moggia et al., 2016b).

### Bruising as Related to Firmness

Firmness is one of the characteristics most frequently measured to evaluate quality of fresh fruit (Timm et al., 1996). As for many other fruit species, firmer blueberries can more readily withstand harvest handling, and will therefore have longer storage potential (Hanson et al., 1993; Yu et al., 2014). Differences in firmness among highbush blueberry cultivars seem to be more dependent on physiological maturity at harvest than on genotypic differences (Beaudry et al., 1998; Lobos et al., 2014a); yet there is limited information on the relevance of firmness at harvest for postharvest quality of fruit within a particular cultivar. Wolfe et al. (1983) demonstrated that firmness separation of blueberries at harvest allows better control of postharvest decay, since soft, medium, and firm fruit show different susceptibility to rot, and fruit segregation enhanced disease control when combined with a hot water dip. Similarly, the present study demonstrates that softening and IB development are related to firmness at harvest of individual fruit, and that high IB can be expected in soft fruit of both cultivars after prolonged storage.

Since in this study the highest IB rates were always found for soft berries (<1.60 N), our findings strengthen the idea that mid-to-firm berries can better withstand a long trip to distant markets. Therefore, any strategy oriented to increase the percentage of these firmness classes into the clamshells will assure higher and more homogeneous quality upon arrival to final destination.

Dropping the fruit did not always lead to higher IB values, and this observation was more evident for “Duke” samples, in which high softening rates but small differences in IB between dropped and non-dropped fruit occurred (**Figure 4**). This finding agrees with the lack of differences between slopes and intercepts of the models fitted for fruit of this cultivar (0 vs. 32 cm drop heights) (**Table 2**); the only difference was found between intercepts of firm fruit, but not between slopes, which indicates similar rates of change in IB per firmness unit both for dropped and non-dropped fruit (**Table 2** and **Figure 5**). Yet, significant associations between firmness and IB, and generally higher *r*^2^ coefficients, both for dropped and non-dropped fruit were obtained for “Duke” as compared to “Brigitta” samples (**Table 2**). On the other hand, the fact that “Brigitta” fruit did not show significant associations for most of the equations indicates a weak relationship between firmness and IB development for this cultivar, especially for samples harvested in Y2. However, higher IB levels in dropped than in non-dropped fruit, regardless of fruit firmness at harvest should be expected for this cultivar (**Table 2** and **Figure 5**). The analyses undertaken for “Brigitta” samples corresponding to Y1 (more heterogeneous in initial condition, and significant *r*^2^ values for dropped fruit uniquely) reveal that differences in slopes and intercepts occurred for all three firmness categories, with different rates of change between dropping treatments. When equations were compared between firmness categories within each dropping treatment (**Table 3**), variability between seasons became more evident, since significances were not the same in both years considered. Moreover, different slopes (meaning dissimilar rate of change in IB per firmness unit) were found on “Duke” 0 cm and “Brigitta” 32 cm, whereas different intercepts (indicating similar rates, but different damage threshold) occurred on “Duke” 32 cm and “Brigitta” 0 cm. Additionally, most of these differences were observed between soft and firm fruit, which emphasizes the negative effects on quality resulting from a high proportion of soft fruit on a particular picking.

According to these results, each cultivar would display a different pattern of IB development when subjected to mechanical damage. Therefore, and depending upon fruit condition at harvest (initial firmness), fruit might not necessarily exhibit severe IB symptoms but would probably show different softening patterns. Another important aspect to consider is that sectioning berries through the equator detects bruising caused by impacts occurring onto that area, but this procedure does not take into account damage at or near the calyx or stem ends, and it would hence lead to an underestimation of the actual mechanical damage (Yu et al., 2014).

The present study demonstrated the different susceptibility to IB development and softening rates in blueberry fruit among different cultivars and firmness categories at harvest, and suggests that fruit displaying firmness lower than 1.6 N at harvest should be avoided if long-term storage is intended. Galletta et al. (1971) proposed that good keeping quality could be expected when TSS/TA ratios are <18, whereas intermediate keeping quality would result from higher TSS/TA values. Given that TSS/TA ratios at harvest of medium and firm fruit ranged from 15 to 21, and that soft fruit values ranged 19–29, it is suggested that this ratio could be used as an additional index to define harvest time and destination of the fruit (long- vs. short-term storage).

Overall, “Duke” fruit were characterized by high rates of firmness loss, as well as by a strong association between firmness and IB, but little differences were found between dropped and non-dropped fruit. “Brigitta” berries had slower softening rates, and displayed very weak relationships between firmness and IB (especially for non-dropped fruit), but marked differences between dropping treatments were found.

## Conclusion

Results of this work suggest that the mean firmness value may be not adequate as an indicator of blueberry fruit condition at harvest, and that the differences in fruit quality traits associated to the initial firmness level might be related to the time that fruit stay on the plant after turning blue, softer fruit displaying more advanced maturity. This finding suggests that, during seasons in which adverse environmental events occur (probably associated to high temperatures close to harvest), the proportion and evolution of soft fruit during shipments would enhance rejections at destination markets. Future research should include a more detailed study on potential sources of fruit heterogeneity. Furthermore, more systematic measurements of changes throughout fruit development from early stages, as done for other species, could help in modeling softening and IB during postharvest. Finally, long-time studies are needed to quantify the real genotypic and environmental effects on softening and IB development in blueberries.

## Author Contributions

CM and GL contributed to the conception and design of the work. GG, CM, and GL performed acquisition, analysis, and interpretation of data for the work. CM, GL, JG, and IL collaborated to generate and validate the version to be published.

## Conflict of Interest Statement

The authors declare that the research was conducted in the absence of any commercial or financial relationships that could be construed as a potential conflict of interest.
