# Supplementary material for: Firmness at Harvest Impacts Postharvest Fruit Softening and Internal Browning Development in Mechanically Damaged and Non-damaged Highbush Blueberries (Vaccinium corymbosum L.)
Source: Front Plant Sci. 2017 Apr 11;8:535. doi: 10.3389/fpls.2017.00535 (PMC5386988; doi:10.3389/fpls.2017.00535)
Supplement: Supplementary file 1 [file Data_Sheet_1.docx]

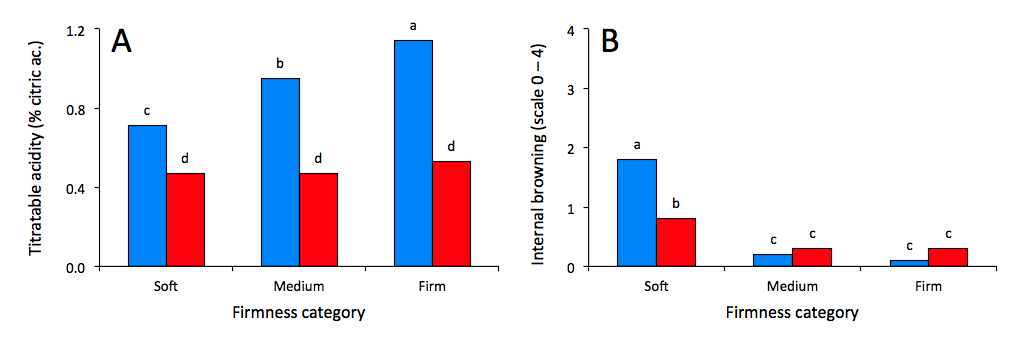
Supplementary Figure 1. Significant interactions of analysis of variance for titratable acidity of ‘Duke’ (A) and internal browning of ‘Brigitta’ blueberries (B) at harvest according, to three firmness category groups, during seasons 2012/13 and 2013/14 (blue and red bars, respectively).
