# Supplementary material for: Firmness at Harvest Impacts Postharvest Fruit Softening and Internal Browning Development in Mechanically Damaged and Non-damaged Highbush Blueberries (Vaccinium corymbosum L.)
Source: Front Plant Sci. 2017 Apr 11;8:535. doi: 10.3389/fpls.2017.00535 (PMC5386988; doi:10.3389/fpls.2017.00535)
Supplement: Supplementary file 2 [file Data_Sheet_2.docx]

Supplementary Table 1. Environmental conditions at two different locations (Longaví and Santa Bárbara) during 2011/12 and 2012/13.
